# Predictive factors for final outcome of severely traumatized eyes with no light perception

**DOI:** 10.1186/1471-2415-12-16

**Published:** 2012-06-19

**Authors:** Rupesh Agrawal, Ho Sue Wei, Stephen Teoh

**Affiliations:** 1 Department of Ophthalmology, Tan Tock Seng Hospital, 11 Jalan, Tan Tock Seng, 308433, Singapore; 2 Ministry of Health Holdings, Singapore, Singapore

**Keywords:** Open globe injury, RAPD, No light perception (NLP), Zone III injury, Vitroretinal trauma

## Abstract

****Background**:**

An eye injury that causes no light perception (NLP) typically carries an unfavorable prognosis, and NLP because of trauma is a common indication for enucleation. With advances in vitreoretinal surgical techniques, however, the indication for enucleation is no longer determined by posttrauma NLP vision alone. There are limited studies in the literature to analyse the outcome of NLP eyes following open globe injury. The current study was aimed to evaluate the outcome of surgical repair of severely traumatized eyes with no light perception vision as preoperative visual acuity. Secondary objective was to possibly predict the factors affecting the final vision outcome in this eyes.

****Methods**:**

Retrospective case analysis of patients with surgical repair of open globe injury over last ten years at a tertiary referral eye care centre in Singapore.

****Results**:**

Out of one hundred and seventy two eyes with open globe injury 27 (15.7%) eyes had no light perception (NLP). After surgical repair, final visual acuity remained NLP in 18 (66.7%) eyes. Final vision improved to Light perception/ Hand movement (LP/HM) in 2(7.4%) eyes, 1/200 to 19/200(11.1%) in 3 eyes and 20/50-20/200(14.8%) in 4 eyes. The median follow up was 18.9 months (range: 4–60 months). The factors contributing to poor postoperative outcome were presence of RAPD (p = 0.014), wound extending into zone III (p = 0.023) and associated vitreoretinal trauma (p = 0.008).

****Conclusions**:**

One third of eyes had ambulatory vision or better though two third of eyes still remained NLP. Pre-operative visual acuity of NLP should not be an indication for primary enucleation or evisceration for severely traumatized eyes. Presence of afferent papillary defect, wound extending posterior to rectus insertion and associated vitreoretinal trauma can adversely affect the outcome in severely traumatized eyes with NLP. Timely intervention and state of art surgery may restore useful vision in severely traumatized eyes.

## **Background**

Ocular trauma can result in a wide spectrum of injury to the eye resulting in mild to severe ocular morbidity. Mechanical injuries to the eye can be classified into open globe injury and closed globe injury [1]. Open globe injury, defined as a full-thickness wound of the eye wall are usually not vision-threatening [1].

There have been numerous variables identified to affect final outcome in patients with open globe injury [2,3]. Those that have been found to correlate significantly with vision outcome include age, type or mechanism of injury, initial visual acuity, presence of a relative afferent pupillary defect (RAPD), extent of wound and size of open-globe injury, location of open globe wound, lens damage, hyphema, vitreous hemorrhage, retinal detachment, and presence of intraocular foreign body [2,3].

An eye injury that causes NLP typically carries an unfavorable prognosis, and NLP because of trauma is a common indication for enucleation [4]. Very few studies have analyzed outcome of severely traumatized eyes with no light perception (NLP). The United States Eye Injury Registry (USEIR) had reported improvement of vision in 16% of eyes with NLP at initial vision [1]. With advances in vitreoretinal surgical techniques, however, the indication for enucleation is no longer determined by posttrauma NLP vision alone.

The current study was aimed to assess the outcome of surgical repair of severely traumatized eyes with no light perception as initial visual acuity following open globe injury. The secondary objective of the study was to possibly predict the preoperative variables affecting the final vision outcome in same group of patients. After obtaining Institutional review board consent to conduct the study, retrospective review of medical records of all the patients with surgical repair following open globe injury were retrieved and analyzed.

## **Methods**

The medical records of 172 patients with consecutive open globe injuries seen at a single tertiary eye centre over 10 year period between 2000 till 2009 were retrospectively reviewed after institutional review board consent. Medical records of all this 172 patients were analyzed in detail. Out of this 172 patients, case records of 27 (15.7%) patients with NLP vision as initial preoperative visual acuity after severe ocular trauma were analyzed in detail for this study. Local Domain Specific Research Board (DSRB) approval was obtained for conducting this retrospective study.

Detailed analysis of the case records included patient demographics, visual acuity at presentation ( in this series it was NLP in all patients), mode of injury, time since injury, ocular tissue/s involved and presence or absence of RAPD, details of operation, number of operations, extent of scleral involvement and final anatomical and visual outcome. All the patients were either local or immigrants in Singapore and industrial accidents or work place related eye injuries was the commonest setting in which eye injury happened. All patients were consulted within six hours of injury due to quite advance healthcare system in developed country like Singapore.

Extent of the wound was classified into zone I, II or III. By definition, zone I injury is corneal wound with wound not extending beyond limbus, zone II injury extend up to 5 mm posterior to limbus into sclera and any injury involving beyond zone II injury i.e., extend of wound beyond 5 mm of sclera from limbus falls into zone III category [1]. Further all the eyes were classified based on international ocular trauma classification system. The parameters which were taken into consideration for classification were mode of injury ( Type B - Blunt or Type A- Penetrating), preoperative visual acuity ( NLP in this series in all patients, Grade 5 as per international classification of open globe injury), presence or absence of relative afferent papillary defect { RAPD – P (positive) or N-(negative)} and zone of injury [1]. Ocular trauma score was also computed for all the patients to predict the possible outcome. Table 1 give detailed distribution of all the 27 patients into different groups based on international ocular trauma classification system and also ocular trauma score at presentation.

**Table 1:** International ocular trauma classification grouping and ocular trauma score for all 27 eyes with no light perception (NLP) following ocular trauma


**S.No**	**Mode of injury**	**Zone of injury**	**RAPD**	**International ocular trauma classification**	**Final VA**	**Raw OTS**	**Final OTS**
1	Penetrating	Zone III	Positive	Type B, Grade 5, Pupil P, Zone III	3	39	1
2	Blunt	zone III	Positive	Type A, Grade 5, Pupil P, Zone III	5	27	1
3	Penetrating	zone II	Positive	Type B, Grade 5, Pupil P, Zone II	5	50	2
4	Blunt	Zone III	Positive	Type A, Grade 5, Pupil P, Zone III	5	27	1
5	Blunt	zone III	Positive	Type B, Grade 5, Pupil P, Zone III	5	27	1
6	Blunt	Zone III	Positive	Type B, Grade 5, Pupil P, Zone III	5	27	1
7	Blunt	zone III	Positive	Type B, Grade 5, Pupil P, Zone III	5	27	1
8	Blunt	zone II	Positive	Type B, Grade 5, Pupil P, Zone II	4	27	1
9	Penetrating	zone II	Positive	Type A, Grade 5, Pupil P, Zone II	5	39	1
10	Penetrating	zone I	Positive	Type A, Grade 5, Pupil P, Zone I	2	50	2
11	Blunt	zone III	Negative	Type B, Grade 5, Pupil N, Zone III	5	6	1
12	Penetrating	zone I	Negative	Type A, Grade 5, Pupil N, Zone I	2	60	2
13	Blunt	zone III	Positive	Type B, Grade 5, Pupil P, Zone III	5	27	1
14	Blunt	zone II	Positive	Type B, Grade 5, Pupil P, Zone II	5	16	1
15	Penetrating	zone III	Positive	Type A, Grade 5, Pupil P, Zone I	3	39	1
16	Penetrating	zone III	Positive	Type A, Grade 5, Pupil P, Zone III	5	50	1
17	Blunt	zone I	Negative	Type B, Grade 5, Pupil N, Zone I	5	37	1
18	Blunt	zone III	Positive	Type B, Grade 5, Pupil P, Zone III	5	27	1
19	Penetrating	zone I	Negative	Type A, Grade 5, Pupil N, Zone I	2	60	2
20	Blunt	zone II	Negative	Type B, Grade 5, Pupil N, Zone II	3	37	1
21	Blunt	zone II	Positive	Type B, Grade 5, Pupil P, Zone II	5	16	1
22	Penetrating	zone III	Positive	Type A, Grade 5, Pupil P, Zone III	4	39	1
23	Blunt	zone III	Positive	Type B, Grade 5, Pupil P, Zone III	5	16	1
24	Blunt	zone III	Positive	Type B, Grade 5, Pupil P, Zone III	5	16	1
25	Blunt	zone III	Positive	Type B, Grade 5, Pupil P, Zone III	5	27	1
26	Blunt	zone II	Positive	Type B, Grade 5, Pupil P, Zone II	5	27	1
27	Blunt	zone III	Positive	Type B, Grade 5, Pupil P, Zone III	2	27	1

Type A: Eyes with penetrating injury.

Type B: Eyes with blunt injury.

Final VA (categorization based on International ocular trauma classification system):

1- 20/40.

2- 20/50-20/200.

3- 1/200-19/200.

4- LP/HM.

5- NLP.

OTS - Ocular trauma score ( United States Eye Injury Registry).

### **Surgical technique**

All the patients underwent primary repair of open globe injuries under general anesthesia. Preoperative consent of very guarded visual prognosis was taken for all the patients. Consent also involved risk of sympathetic ophthalmia, multiple surgeries if required, and risk for enucleation or evisceration of the globe in case of unsalvageable eye.

None of the eyes underwent primary enucleation or evisceration. Under general anaesthesia, standard surgical steps involved 360 degrees conjunctival peritomy with blunt dissection of conjunctiva. Limbal laceration if present was identified and secured first with 10-0nylon suture. It was followed by corneal laceration repair with interrupted 10-0nylon sutures. Scleral laceration was subsequently explored and sutured with interrupted 7–0 vicryl sutures. For scleral wound extending beyond rectus insertion, the rectus was disinserted after placing preplaced 6-0vicryl suture and clamping the muscle tendon at insertion with artery clamp before cutting to prevent bleeding. Total extent of the scleral wound was explored and sutured. Care was taken to prevent incarceration of the uveal/choroidal tissue or retinal tissue into the scleral wound. Prolapsing vitreous was also cut with help of Vennas scissors and sponge. After securing watertight closure of scleral laceration, disinserted muscle was secured back to its insertion site. Generous subtenon’s antibiotic wash was given before closure of peritomy. Corneal laceration and limbal laceration was reexamined for its integrity and after forming anterior chamber with Balanced Salt Solution (BSS), wound was reexamined with sterile fluorescein drops for any leak by Siedel’s test technique. On subsequent postoperative evaluation, clinical status of the eye including visual acuity, anterior and posterior segment examination and serial B-scans for posterior segment status was carried out. Second stage vitreoretina surgery with or without lens extraction was performed for further vision rehabilitation at a later stage in indicated eyes. Three port pars plana vitrectomy was performed after one to two weeks of primary wound repair. Total vitreous gel removal with posterior vitreous detachment induction and base excision was performed in all cases undergoing vitrecomy. For eyes with retinal detachment and vitreoretinal incarceration, relaxing retinotomy and if required retinectomy was performed. 360 degree endolaser was performed in all vitrectomised eyes and silicone oil was used as tamponading agent.

## **Results**

Mean age was 39.6 ± 18.6 (Range: 15–91) yrs with a male predominance of 85.2%. Mean follow up was 18.9 months (Range: 4–60 months) and median follow up was 16 months. There were 18 (67.7%) eyes that sustained blunt injury and 9 (33.3%) eyes with penetrating injury. There were 4 (14.8%) eyes with injury involving zone I, 7 (25.9%) eyes with injury involving zone II and 16 (59.2%) eyes with wound extending into zone III.

Associated ocular structures involved in injury are summarized in Table 2. The most common included hyphema (100%), vitreous hemorrhage (77.8%) and traumatic cataract (70.4%). Intraoperatively scleral laceration was found to be extending beyond rectus insertion in 16 (59.25%) eyes. Nineteen (70.4%) eyes underwent one surgery, 7 (25.9%) eyes had two surgeries and 1 (3.7%) eye had three surgeries. None of the eyes underwent enucleation or evisceration. Second and subsequent surgeries were for associated vitreoretinal trauma and associated traumatic cataract. Final vision outcome remained NLP in 18 (66.7%) eyes. However, final vision improved to light perception hand movement (LP/HM) in 2 (7.4%) eyes, 1/200 to 19/200 in 3 (11.1%) eyes and 20/50-20/200 in 4 (14.8%) eyes. Anatomically, out of total 27 eyes, six (22.22%) eyes had phthisical changes.

**Table 2:** Ocular tissue involved in eyes with open globe injury with NLP


**Ocular tissue involved**	**No (%) of eyes**
Orbital involvement	7 (25.9%)
Relative afferent pupillary defect	22 (81.5%)
Traumatic cataract	19 (70.4 %)
Hyphaema	27 (100%)
IOFB	3 (11.1%)
Vitreous loss	12 (44.4 %)
Vitreous Hemorrhage	21 (77.8%)
Retinal detachment	10 (37.0%)
Lid laceration	13 (48.1%)

### **Statistical analysis**

Statistical tests were applied to assess the correlation between variables associated with traumatized globe. Spearman’s rho correlation analysis was applied to study the correlation between different variables (Table 3 and Table 4). Based on Spearman’s rho correlation analysis and Chi square test and by applying Fischer’s exact test for ordinal-categorical and categorical- categorical data, there was a statistical significant association between zone of injury and RAPD (p = 0.014), zone & VA (p = 0.023), mode of injury & VA (p = 0.022), mode of injury & vitreous hemorrhage (p = 0.008), associated vitreoretinal trauma and final VA (p = 0.008) and also more number of operations positively affecting final vision outcome (p = 0.011). Similarly, eyes with zone III injury were statistically more associated with RAPD and poor visual acuity. (p = 0.014 based on Fisher’s test of independence after using Chi square test). Open globe injury with blunt trauma was more significantly associated with vitreous hemorrhage (p = 0.008 based on Fisher’s test of independence after using Chi square test (Table 5).

**Table 3:** Spearman’s rho correlation analysis


**Spearman’s rho correlation analysis**		**Age**	**Sex**	**Eye**	**Zone**	**Mode of injury**	**Lid involvement**	**Orbital involvement**	**RAPD**
Age	Correlation coefficeint	1.00	.59	-.04	-.09	.16	.00	-.08	.39 (*)
	Sig. (2-tailed)	.	.001	.84	.631	.406	.981	.666	.043
Sex	Correlation Coefficient	.597(**)	1.000	.147	-.196	.295	-.193	-.009	.199
	Sig. (2-tailed)	.001	.	.463	.326	.135	.334	.965	.320
Eye	Correlation Coefficient	-.040	.147	1.000	.051	.167	-.210	.120	.135
	Sig. (2-tailed)	.841	.463	.	.800	.406	.294	.553	.502
Zone	Correlation Coefficient	-.097	-.196	.051	1.000	.063	.183	.374	.566(**)
	Sig. (2-tailed)	.631	.326	.800	.	.756	.362	.055	.002
Mode of Injury	Correlation Coefficient	.167	.295	.167	.063	1.000	.210	.239	.067
	Sig. (2-tailed)	.406	.135	.406	.756	.	.294	.230	.738
Lid involvement	Correlation Coefficient	.005	-.193	-.210	.183	.210	1.000	.106	.078
	Sig. (2-tailed)	.981	.334	.294	.362	.294	.	.597	.700
Orbital Involvement	Correlation Coefficient	-.087	-.009	.120	.374	.239	.106	1.000	.064
	Sig. (2-tailed)	.666	.965	.553	.055	.230	.597	.	.749
RAPD	Correlation Coefficient	.393(*)	.199	.135	.566(**)	.067	.078	.064	1.000
	Sig. (2-tailed)	.043	.320	.502	.002	.738	.700	.749	.
Lens Status	Correlation Coefficient	.355	.291	.136	-.315	-.172	-.123	-.210	-.036
	Sig. (2-tailed)	.069	.141	.498	.109	.392	.541	.292	.859
IOFB	Correlation Coefficient	.038	-.147	.000	.418(*)	-.500(**)	-.105	.060	.169
	Sig. (2-tailed)	.851	.463	1.000	.030	.008	.603	.767	.401
Vitreous Loss	Correlation Coefficient	-.192	-.373	.000	.162	.158	.033	-.189	.235
	Sig. (2-tailed)	.338	.055	1.000	.419	.431	.870	.345	.239
Vitreous Hemorrhage	Correlation Coefficient	.103	.223	.189	.219	.567(**)	.158	.316	.204
	Sig. (2-tailed)	.609	.264	.345	.271	.002	.430	.108	.308
Retinal Detachment	Correlation Coefficient	-.424(*)	-.320	-.054	.500(**)	-.108	.028	.421(*)	.168
	Sig. (2-tailed)	.027	.104	.788	.008	.590	.888	.029	.402
Wound extending Posterior to Rectus Insertion	Correlation Coefficient	.121	.079	.053	.180	.107	.106	.197	.201
	Sig. (2-tailed)	.547	.697	.792	.368	.597	.598	.323	.314
Number of Operations	Correlation Coefficient	-.088	-.050	-.127	-.293	-.418(*)	.048	-.204	-.092
	Sig. (2-tailed)	.664	.803	.529	.138	.030	.813	.307	.647
Final Visual Acuity	Correlation Coefficient	.046	.104	-.121	.546(**)	.508(**)	.274	.410(*)	.308
	Sig. (2-tailed)	.818	.604	.548	.003	.007	.167	.034	.118

**Table 4:** Spearman’s rho correlation analysis

**Spearman’s rho correlation analysis**		**IOFB**	**Vitreous loss**	**Vitreous hemorrhage**	**Retinal detachment**	**Wound extending posterior to recti**	**Number of operations**	**Final visual acuity**
Age	Correlation Coefficient	.03	−0.19	.10	−0.42(*)	.12	−0.08	.04
	Sig. (2-tailed)	.851	.338	.609	.027	.547	.664	.818
Sex	Correlation Coefficient	−0.147	−0.37	.223	−0.320	.079	−0.050	.104
	Sig. (2-tailed)	.463	.055	.264	.104	.697	.803	.604
Eye	Correlation Coefficient	.000	.000	.189	−0.054	.053	−0.127	−0.121
	Sig. (2-tailed)	1.000	1.000	.345	.788	.792	.529	.548
Zone	Correlation Coefficient	.418(*)	.162	.219	.500(**)	.180	-.293	.546(**)
	Sig. (2-tailed)	.030	.419	.271	.008	.368	.138	.003
Mode of Injury	Correlation Coefficient	-.500(**)	.158	.567(**)	-.108	.107	-.418(*)	.508(**)
	Sig. (2-tailed)	.008	.431	.002	.590	.597	.030	.007
Lid involvement	Correlation Coefficient	-.105	.033	.158	.028	.106	.048	.274
	Sig. (2-tailed)	.603	.870	.430	.888	.598	.813	.167
Orbital Involvement	Correlation Coefficient	.060	-.189	.316	.421(*)	.197	-.204	.410(*)
	Sig. (2-tailed)	.767	.345	.108	.029	.323	.307	.034
RAPD	Correlation Coefficient	.169	.235	.204	.168	.201	-.092	.308
	Sig. (2-tailed)	.401	.239	.308	.402	.314	.647	.118
Lens Status	Correlation Coefficient	.160	-.017	-.074	-.231	-.193	-.036	-.284
	Sig. (2-tailed)	.426	.933	.714	.246	.334	.859	.151
IOFB	Correlation Coefficient	1.000	-.316	-.094	-.027	-.053	-.228	.245
	Sig. (2-tailed)	.	.108	.639	.893	.792	.253	.218
Vitreous Loss	Correlation Coefficient	-.316	1.000	.120	.240	.017	-.060	-.011
	Sig. (2-tailed)	.108	.	.553	.228	.933	.766	.955
Vitreous Hemorrhage	Correlation Coefficient	-.094	.120	1.000	.041	.081	-.215	.384(*)
	Sig. (2-tailed)	.639	.553	.	.839	.689	.281	.048
Retinal Detachment	Correlation Coefficient	-.027	.240	.041	1.000	-.012	.198	.124
	Sig. (2-tailed)	.893	.228	.839	.	.954	.323	.538
Wound extending Posterior to Rectus Insertion	Correlation Coefficient	-.053	.017	.081	-.012	1.000	-.170	.122
	Sig. (2-tailed)	.792	.933	.689	.954	.	.396	.545
Number of Operations	Correlation Coefficient	-.228	-.060	-.215	.198	-.170	1.000	-.528(**)
	Sig. (2-tailed)	.253	.766	.281	.323	.396	.	.005
Final Visual Acuity	Correlation Coefficient	.245	-.011	.384(*)	.124	.122	-.528(**)	1.000
	Sig. (2-tailed)	.218	.955	.048	.538	.545	.005	.

**Table 5:** Spearman’s rho correlation analysis and Chi square test and by applying Fischer’s exact test for ordinal-categorical and categorical-categorical data

**Correlation between variables**	**Fischer’s exact test:**
Zone III injury and RAPD	0.014
Zone III injury and poor final VA	0.023
Blunt injury and vit hemorrhage	0.008
Blunt injury and poor final VA	0.022
More # of operations and good final VA	0.011

Also, presence of intraocular foreign body was not statistically associated with adverse outcome in our current study. All the patients with intraocular foreign bodies were operated within six hours of injury and hence had no impact of delaying the surgery with retained intraocular foreign body. There were no cases of post traumatic endophthalmitis as well in the current study.

Predictability of Ocular trauma score was also correlated with outcome in this 27 eyes and it was found to be having close correlation between our study group and Ocular trauma score prediction of final vision outcome [5]. However, because of small sample size , statistical tests to establish the significance of same could not be applied (Table 6).

**Table 6:** **Correlation of Ocular trauma score in our current study with ocular trauma score by United States Eye Injury Registry **[5]

**Raw OTS**	**OTS**	**NLP**	**LP/HM**	**1/200-**	**20/200-**	**>20/40**
				**19/200**	**20/50**	
0-44	1 (23 eyes)	17 eyes	2 eyes (8.6%)	3 eyes	1 eye (4.3%)	0 eye (0%)
	USEIR OTS	(73.9%) 73%	17%	(13.0%) 7%	2 %	1%
45-65	2 (4 eyes)	1 eye	0 eye	0 eyes	3 eye	0 eye
	USEIR OTS	(25%) 28%	(0%) 26%	(0%) 18%	(75%) 13%	(0%) 15%
66-80	3	0 (0%)	0 (0%)	0 (0%)	0 (0%)	0 (0%)
81-91	4	0 (0%)	0 (0%)	0 (0%)	0 (0%)	0 (0%)
92-100	5	0 (0%)	0 (0%)	0 (0%)	0 (0%)	0 (0%)

## **Discussion**

Severely traumatized eyes with extensive corneoscleral laceration render primary repair of the globe challenging. In eyes with NLP vision and severe corneoscleral lacerations, repair of the globe is even more challenging and prognosis is often guarded.

The outcome of surgical management of injured eyes with severe vision loss had been reported with different success rates. In data from USEIR, all the eyes with initial visual acuity of hand movements or better either improved from presenting visual acuity or maintained same vision [1]. Ebrahimi et al. [6] reported the anatomical and visual outcomes of surgical repair on 12 injured eyes with Grade 4 RAPD; 11 had successful results including visual acuity between hand motion and 20/40, attached retina, and normal intraocular pressure. Morris et al. [4] reported favorable vision outcome in severely traumatized eyes with no light perception with opaque media with one eye reaching 20/40 visual acuity, and another reaching 20/100 visual acuity postoperatively. In another study of nineteen eyes with no light perception vision with severe ocular trauma by Heidari et al. [7] final visual acuity improved to LP in 3 eyes, hand motion in 4 eyes, counting fingers in 3 eyes, and 20/200 in 7 other eyes. In their study, chi-square test showed no significant association between age, sex, type or location of injury, lens status, hyphema, endophthalmitis, IOFB or RD, and final best-corrected visual acuity. Salehi-Had et al. [8] reported eight NLP cases after OGI that received vitreoretinal surgery. Five had vision improvement ranging from hand movement to 20/70; 2 had a stable vision of LP; and 1 eye returned to NLP. Schmidt et al. [9] reported on 39 eyes with NLP in which 6 eyes recovered more than LP vision.

In the current study, we had 33.3% of the eyes recovery to light perception or better. Preperative variables such as hyphaema, traumatic cataract and dense vitreous hemorrhage possibly could account for for no light perception vision in this group of patients. Similarly, eyes with post traumatic retinal detachmentwith NLP had improvement in vision following surgical intervention. Preoperative variables such as RAPD ,posteriorly extending wound beyond insertion of rectus muscle and significant vitreoretinal trauma were factors associated with adverse outcome. Associated vitreoretinal trauma with vitreous loss and retinal tissue prolapse out of the scleral laceration accounted for poorer vision outcome (p = 0.008). In a very recent case control study by Feng et al, authors pointed out that ciliary body damage, closed funnel retinal detachment, and choroidal damage are independent risk factors for NLP posttrauma but not prognostic indicators for NLP visual outcome [10]. They concluded that traumatized eyes with NLP may recover light perception or better vision if appropriate interventional measures are used for treatment of the injured ciliary body, retina, and choroid [10]. In our study, number of operations had statistically significant positive effect on the final vision outcome as second operation was performed for removal of media opacity or repair of retinal detachment which were contributing for preoperative visual acuity of no light perception.

Visual acuity can be profoundly impaired to the extent of NLP in presence of significant media opacity (e.g. corneal edema, hyphema, cataract, dense vitreous hemorrhage), retinal detachment, associated subretinal or subhyaloid hemorrhage, hemorrhagic choroidals and even psychological factors (e.g. hysteria). Assessment of light perception is a subjective measure and not a fool proof test in the presence of severe media opacity secondary to dense vitreous hemorrhage, traumatic cataract, dense hyphema and corneal edema. Assessment of light perception even with the bright light of an indirect ophthalmoscope can give false impression of NLP [11]. RAPD is an indicator of damage to the optic nerve or retina, it may be falsely positive in the presence of severe hyphema or subretinal vitreous hemorrhage and may disappear after resorption or removal of the hemorrhage [12]. Ultrasonography is useful for assessment of posterior segment in the eyes with media opacity and to differentiate between retinal detachment and vitreous hemorrhage, but it is sometimes difficult to differentiate a detached retina from blood clots in the vitreous cavity or membranes [13].

Before deciding on enucleation in patients with NLP, reversible causes of vision loss should be excluded including psychological factors [4,14]. Even in situations in which enucleation seems inevitable, the ophthalmologist should discuss the possible options with the patient before making a final decision. Primary enucleation for severely traumatized eyes with NLP in view of risk of sympathetic ophthalmia was a controversial approach [14]. Sympathetic ophthalmia with potential for bilateral blindness is a relative indication for enucleation of an injured eye [4,14]. Most reported cases (65%) occur between 2 weeks to 2 months after injury and is rare during the first 2 weeks after trauma [15]. However the actual rate of post-traumatic sympathetic ophthalmia is not clear, and reported rates vary from 0.28% to 1.9% [4,15]. The use of modern immunosuppressives has also improved treatment and control of sympathetic ophthalmia [4]. As such primary surgical repair should not be abandoned for the risk of sympathetic ophthalmia in eyes with NLP.

Visually evoked potential (VEP) and bright light flash electroretinography are important ancillary measures for making a preoperative decision, but these tests are not completely reliable either. Though none of the patients in our series underwent flash VEP, based on literature review non-recordable VEP or brightlight flash electroretinography in the presence of dense vitreous hemorrhage does not necessarily indicate that the visual potential is lost. It has been shown that both electroretinography and VEP are reduced in the presence of dense vitreous hemorrhage [10,16].

Current study had few limitations. Because of retrospective design of the study, data collection is always limited and there is always some missing data which is one of the inherent bias in retrospective studies. Assessment of perception of light was not standardized. Definitive tools of optic nerve assessment like visual evoked Potential was not performed in the current study. Similarly, small number of subjects from single centre has its own set of limitations in predicting study outcome.

As there are no reliable indirect tests to prognosticate the final vision outcome in presence of significant media opacity, the decision for primary enucleation or evisceration in recently injured eyes with severe media opacity is difficult to predict at the outset. The only practical way may be direct observation of vital structures of the injured eye during surgical exploration. Clearing the hyphema and the vitreous hemorrhage together with cataract extraction where necessary during exploration may enable the surgeon to determine whether further surgery is warranted to evaluate the visual prognosis.

## **Conclusions**

In our study, almost two third of patients regained vision of light perception or better with one third of patients improving to visual acuity of 6/45 or better. It highlights the fact that even in severely traumatized eyes with NLP, surgical repair can restore ambulatory vision. None of the patients in the series developed sympathetic ophthalmia and none underwent primary enucleation. Preservation of the globe offers the advantage of positive psychological impacts on the patient and his relatives. As discussed earlier, the patient may be the only one who can decide to undergo reparative surgery in eyes with no light perception and accept the risk of sympathetic ophthalmia with limited vision prognosis. Counselling of the trauma victim and family forms an integral part in management of severely traumatized eyes. Most patients may prefer to retain the repaired eye because of the great psychological impact of enucleation [17]*.*

The surgeon’s role is to help the patient make the best decision in this regard. However, larger multicentre prospective studies are recommended to assess the final anatomical and visual outcome in eyes with no light perception following surgical intervention in open globe injury.

## **Competing interests**

There are no competing interest for any authors for this manuscript.

## **Authors’ contributions**

Dr SWH:- contributed for data collection. Dr RA:- Data analysis and manuscript compilation. Dr ST:- Manuscript review and critical remarks, mentor. All authors read and approved the final manuscript.

## Pre-publication history

The pre-publication history for this paper can be accessed here:

http://www.biomedcentral.com/1471-2415/12/16/prepub
